# Synthesis and Anticancer Evaluation of New 1,3,4-Oxadiazole Derivatives

**DOI:** 10.3390/ph14050438

**Published:** 2021-05-06

**Authors:** Camelia Elena Stecoza, George Mihai Nitulescu, Constantin Draghici, Miron Teodor Caproiu, Octavian Tudorel Olaru, Marinela Bostan, Mirela Mihaila

**Affiliations:** 1 Department of Pharmaceutical Chemistry, Faculty of Pharmacy, “Carol Davila” University of Medicine and Pharmacy, 6 Traian Vuia Street, 020956 Bucharest, Romania; camelia.stecoza@umfcd.ro (C.E.S.); octavian.olaru@umfcd.ro (O.T.O.); 2 “Costin D. Neniţescu” Centre of Organic Chemistry Romanian Academy, 202 B Splaiul Independenţei, 060023 Bucharest, Romania; cst_drag@yahoo.com (C.D.); mct@ccocdn.ro (M.T.C.); 3 Center of Immunology, “Stefan S. Nicolau” Institute of Virology, 030304 Bucharest, Romania; marinela.bostan@virology.ro (M.B.); mirela.mihaila@virology.ro (M.M.)

**Keywords:** cytotoxic agents, apoptosis induction, HT-29 cells, MDA-MB-231 cells, mechanism prediction, STAT inhibitors, miR-21, hydrazide derivatives, nitrogen scaffolds

## Abstract

In order to develop novel chemotherapeutic agents with potent anticancer activities, a series of new 2,5-diaryl/heteroaryl-1,3,4-oxadiazoles were designed and synthesized. The structures of the new compounds were established using elemental analyses, IR and NMR spectral data. The compounds were evaluated for their anticancer potential on two standardized human cell lines, HT-29 (colon adenocarcinoma) and MDA-MB-231 (breast adenocarcinoma). Cytotoxicity was measured by MTS assay, while cell cycle arrest and apoptosis assays were conducted using a flow cytometer, the results showing that the cell line MDA-MB-231 is more sensitive to the compounds’ action. The results of the predictive studies using the PASS application and the structural similarity analysis indicated STAT3 and miR-21 as the most probable pharmacological targets for the new compounds. The promising effect of compound **3e**, 2-[2-(phenylsulfanylmethyl)phenyl]-5-(4-pyridyl)-1,3,4-oxadiazole, especially on the MDA-MB-231 cell line motivates future studies to improve the anticancer profile and to reduce the toxicological risks. It is worth noting that **3e** produced a low toxic effect in the *D. magna* 24 h assay and the predictive studies on rat acute toxicity suggest a low degree of toxic risks.

## 1. Introduction

Cancer, a severe human health issue, is among the leading causes of death on a global scale, and so far chemotherapy remains a main treatment option adopted worldwide either alone or in conjunction with surgery and/or radiotherapy [1]. Despite the significant advancement in chemotherapy over recent decades, which led to major changes in the treatment of various cancers, the main obstacles to the success of the therapy have remained the development of tumor-cell resistance to various chemotherapeutic agents. Drug resistance, either existing before treatment (intrinsic) or generated after therapy (acquired), is responsible for most the relapses of cancer, one of the major causes of death of the disease [2,3].

**Figure 1:**
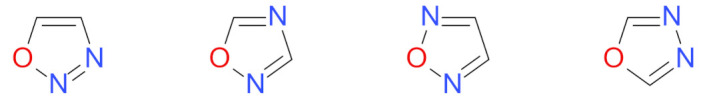
Chemical structures of oxadiazole isomers.

In the attempt of finding effective anticancer agents, the strategy of using simple druggable scaffolds proved to be successful in finding many relevant lead compounds [4]. A large number of nitrogen-containing heterocyclic compounds were identified as valuable anticancer solutions [5]. Among these, oxadiazoles are receiving particular interest. Depending on the position of the nitrogen and oxygen atoms, the heterocycle may occur in the form of one of the following four different isomers: 1,2,3-, 1,2,4-, 1,2,5-, and 1,3,4-oxadiazole (Figure 1) [6].

The greatest interest is involved with 1,3,4-oxadiazoles, as in the last years a large number of compounds with cytotoxicity for several tumor lines have been reported. The most active derivatives are more potent than the reference drugs, which proves the high anticancer potential of the 1,3,4-oxadiazole ring [7,8,9,10,11]. The 1,3,4-oxadiazole is an important scaffold in medicinal chemistry, with high versatility, giving rise to elevated structural diversity. In some cases, it acts as a bioisostere for carbonyl-containing compounds such esters, amides, and carbamates or as a flat aromatic linker to provide the appropriate molecular geometry. The stability of the oxadiazole ring in aqueous medium, and its capability to easily interact with bio-targets establishing π –π interactions or forming strong hydrogen bonds, justify the interest in the development of bioactive molecules containing this scaffold [6].

**Figure 2:**
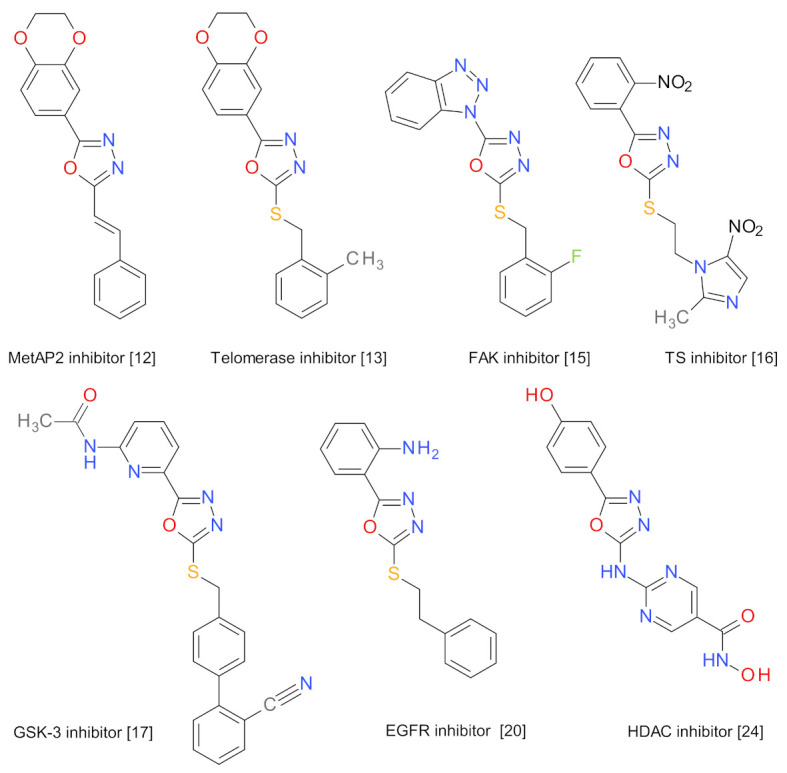
Structure of some representative bioactive compounds containing 1,3,4-oxadiazole moiety as anticancer agents.

The versatility and the usefulness of the 1,3,4-oxadiazole scaffold is demonstrated by its use as a core structure in the inhibitors of methionine aminopeptidase (MetAP2) [12], telomerase [13,14], focal adhesion kinase (FAK) [15], thymidylate synthase (TS) [16], glycogen synthase kinase-3 (GSK-3) [17], and thymidine phosphorylase (TP) [18,19]. The antitumor potency of 1,3,4-oxadiazoles derivatives is also related to their ability to inhibit grow factors such as epidermal growth factor receptor (EGFR) [20,21] or vascular endothelial growth factor (VEGF) [22], to inhibit tubulin polymerization [23], histone deacetylases (HDAC) [24,25], or to interact with DNA structures [26]. The structures of the representative anticancer compounds sharing the 1,3,4-oxadiazole scaffold are presented in Figure 2.

In view of the above findings, in order to develop novel chemotherapeutic agents with potent anticancer activities we hereby report the synthesis, characterization and biological evaluation of some new 2,5-diaryl/heteroaryl-1,3,4-oxadiazoles.

## 2. Results

### 2.1. Synthesis Procedures

The compounds were designed so that the molecular weight is under 500 g/mol, and the number of hydrogen donors and acceptors comply with the Lipinski rule.

**Scheme 1:**
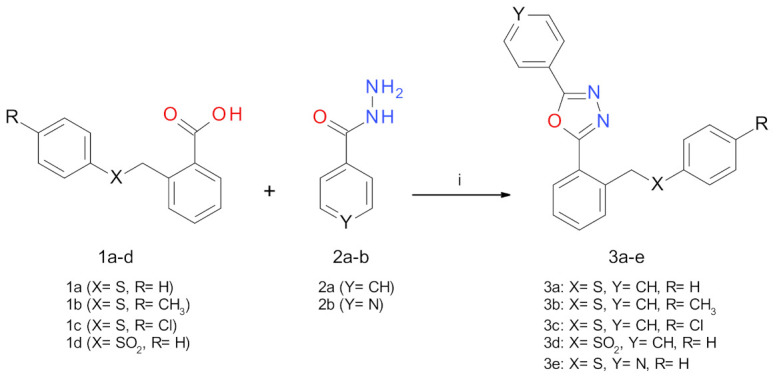
Synthesis of the target compounds **3a–e**. Reagents and conditions: (i) POCl_3_, reflux, 9 h.

The new 1,3,4-oxadiazole derivatives were prepared by heating under reflux and magnetic stirring of aromatic carboxylic acids **1a–d** with the hydrazide derivatives **2a–b** in the presence of phosphorus oxychloride in 59–70% yields (Scheme 1).

The aromatic carboxylic acids **1a–c** were prepared starting from thiophenol or the corresponding *p*-substituted thiophenols and phtalide, according to a previously reported procedure [27,28]. A synthetic procedure for the preparation of 2-[(benzenesulfonyl)methyl]benzoic acid (**1d**) was reported by Patra group [29], and it consists in the reaction of methyl 2-(bromomethyl)benzoate with sodium benzenesulfinate in dry dimethylformamide at room temperature, followed by the hydrolysis of the resulting ester with an aqueous solution of sodium hydroxide. The benefit of our synthesis method is the use of an alternative greener approach and the yield advantage.

The new 1,3,4-oxadiazole derivatives **3a–e** were prepared by the treatment of aromatic carboxylic acids **1a–d** with the hydrazide derivatives **2a–b** in the presence of phosphorus oxychloride. The method was adapted based on previously described procedures [30,31].

**Scheme 2:**
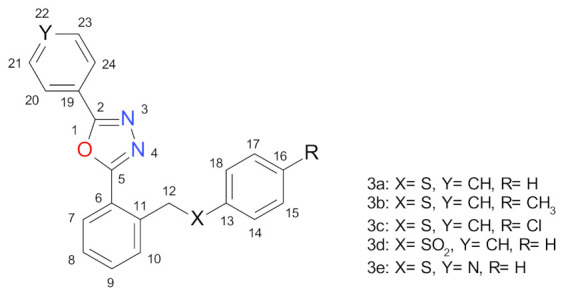
Structure of the new oxadiazole compounds and the numbering of their atoms.

Scheme 2 presents the atoms’ numbering used for assigning the NMR signals of the new oxadiazole derivatives.

In the ^1^H-NMR spectra of the new compounds, the aromatic hydrogens gave signals in the range of 8.84–6.98 ppm. The methylene group CH_2_ (H-12) presented a singlet in the range of 4.64–4.71 ppm for the compounds **3a–c** and **3e**. In the case of **3d**, the S-oxidation induces a deshielding effect that results in an approximately 1 ppm higher chemical shift for the protons of the methylenic group. The methyl group presented a singlet at 2.22 ppm.

In the ^13^C-NMR spectra, the two signals in the range of 164.50–161.87 ppm are produced by the two carbons in the oxadiazole ring. The carbon atoms of the benzene and the pyridine rings produce signals in the range of 149.80–120.41 ppm. The methylene group (C-12) is characterized by a signal at 36.54–38.70 ppm in the compounds **3a–c** and **3e**, and 59.38 ppm in compound **3d**.

The IR spectra of the **3a–e** compounds differ significantly from the corresponding spectra of the hydrazide derivatives **2a–b** and those of the acids **1a–d**.

### 2.2. Anticancer Evaluation

The amplification of the cell division process is responsible for the formation of tumors. Most tumor cells have disorders in the development of the cell cycle, which are associated with an exacerbated proliferative process and this is responsible for the evolution of the tumor process. In addition, the apoptotic process is inhibited in tumor cells [32,33]. In general, cytostatic treatment aims either to induce the apoptotic process of the tumor cells or to cause cell cycle blockage.

To determine the role of the studied compounds on tumor processes, studies were performed on two different types of cancer (colon and breast) using the standardized cell lines HT-29 and MDA-MB-231. The cells treated with the compounds **3a–e** and the intermediates **2a–b** were subjected to flow cytometry techniques in order to examine the apoptotic process and the cell cycle analysis. The apoptotic cells were determined by flow cytometry using Annexin V-FITC and PI (propidium iodide) double labeling. The live cell population, and the cells undergoing early apoptosis (Annexin+/PI−) and late apoptosis (Annexin+/PI+) were quantified [34,35]. The distribution of the cell cycle phases in the tumor cells treated for 24 h with the studied compounds was analyzed for their DNA content by flow cytometry [36,37].

Cisplatin (CisPt) is frequently used for the treatment of colon adenocarcinoma, while doxorubicin (DOX) is routinely used in the treatment of breast cancers, and was therefore chosen as a reference (CisPt for HT-29 cells, DOX for MDA-MB-231 cells).

#### 2.2.1. Effects on Cell Viability

The cell viability after treatment with the compounds and reference drugs was determined using the MTS (3-(4,5-dimethylthiazol-2-yl)-5-(3-carboxymethoxyphenyl)-2-(4-sulfophenyl)-2H-tetrazolium). The HT-29 and MDA-MB-231 tumor cells were preliminarily treated with the new compounds in concentrations from 6.25 µM up to 200 µM for 24 or 48 h in order to determine the optimal concentration. Two concentrations, 10 μM and 50 μM, were chosen to evaluate the compounds effect on the cells’ viability.

**Figure 3:**
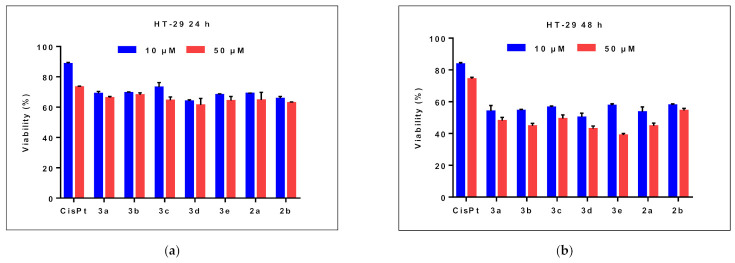
The tested compounds effects on HT-29 tumor cells’ viability. The cells were treated with the compounds compared with cisplatin (CisPt) using two concentrations 10 µM or 50 µM for 24 h (**a**) or 48 h (**b**). Untreated cells were considered to have 100% viability.

**Figure 4:**
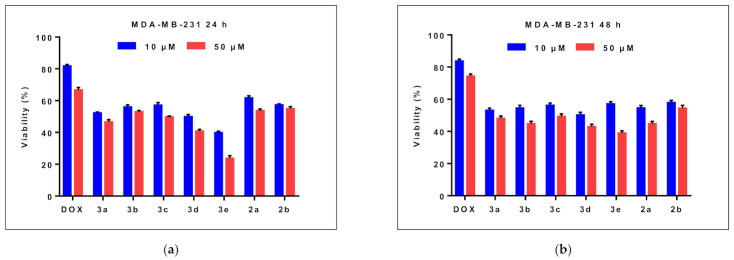
The tested compounds effects on MDA-MB-231 tumor cells’ viability. The cells were treated with the compounds compared with doxorubicin (DOX) using two concentrations 10 µM or 50 µM for 24 h (**a**) or 48 h (**b**). Untreated cells were considered to have 100% viability.

The two cell lines responded differently to treatment with the tested compounds. In the HT-29 cell line, the tested compounds reduce viability in a similar way regardless of the concentration used (Figure 3). In contrast, in the MDA-MB231 line, the cell viability is affected more when the compounds are used in a higher concentration (50 μM) and the chemical structure has a greater impact, the compound **3e** having the strongest effect (Figure 4). It is noteworthy that all the compounds analyzed have a stronger cytotoxic effect than the effect induced by CisPt or DOX.

The new oxadiazole derivatives **3a–e** reduced the HT-29 cells’ viability with values in the range of 64.0% (**3d**) up to 73.2% (**3c**) when exposed for 24 h at 10 µM, and with values between 61.5% (**3d**) and 68.1% (**3b**) when the concentration was 50 µM. The effect of the compounds was higher when the exposure was doubled to 48 h. The cells’ viability was between 50.3% (**3d**) and 57.7% (**3e**) for 10 µM, and between 39.0% (**3e**) and 49.4% (**3c**) for the 50 µM concentration.

The compounds **3a–e** reduced the MDA-MB-231 cells’ viability with values in the range of 39.9% (**3e**) up to 57.2% (**3c**) when exposed for 24 h at 10 µM. The viability values were between 23.8% (**3e**) and 53.1% (**3b**) when the concentration was 50 µM. The cells’ viability was between 50.8% (**3d**) and 56.9% (**3e**) after exposure for 48 h at 10 µM, and between 38.7% (**3e**) and 48.9% (**3c**) for the 50 µM concentration.

#### 2.2.2. Effects on Cell Apoptosis

The HT-29 and MDA-MB-231 cells were treated for 24 h with the compounds **3a–e** and the intermediates **2a–b,** and subjected to a double Annexin/PI staining technique that allowed the detection of the apoptotic process by flow cytometry. CisPt and DOX were used as positive controls (Table 1).

The treatment of the HT-29 cells for 24 h with 10 µM of the compounds **3a–e** increased the total apoptosis in the range of 9.4% up to 51.2% compared to the untreated cells (control, 5.9 %). Considering the compound **3a**, the introduction of a 4-chloro substituent is detrimental for the apoptotic effect (compound **3c**), while the transformation of the sulfur atom into a sulfone (compound **3d**) slightly reduced the effect. The exchange of the benzene ring with a pyridine (compound **3e**) almost doubled the proportion of apoptotic cells.

**Figure 5:**
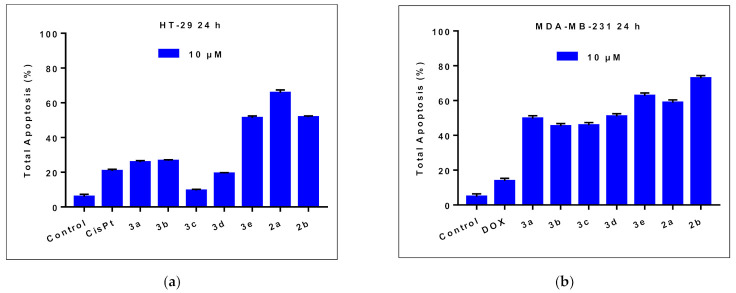
The tested compounds effects on cells’ apoptosis. The cells were treated with the test compounds at 10 µM for 24 h. The effects on HT-29 cells can be observed compared to cisplatin (CisPt) (**a**) and the effects on MDA-MB-231 cells are reported to doxorubicin (DOX) (**b**).

The new oxadiazole compounds had greater effects on the MDA-MB-231 cells compared to those observed on the HT-29 cells, with the total apoptosis percent’s in the range of 45.2% up to 62.7%. All the compounds had close to three-fold stronger effects than those of the positive control. The observed structure activity relationships are similar, but the impact of the structural transformation is smaller. The compound **3e** determined the greatest effect in the oxadiazoles series, but it was smaller when compared with its precursor hydrazide **2b** (Figure 5).

#### 2.2.3. Cell Cycle Analysis

**Figure 6:**
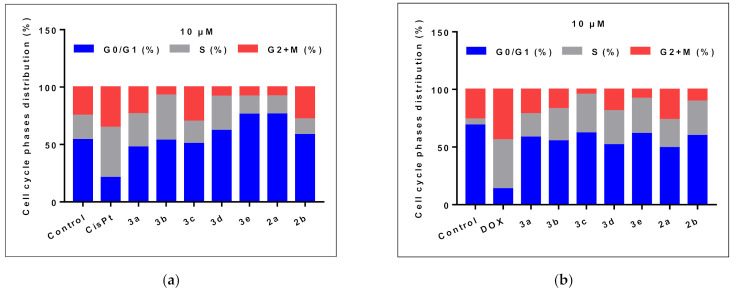
The tested compounds’ effects on cell cycle phases. The cells were treated with the test compounds at 10 µM for 24 h. The effects on HT-29 cells can be observed compared to cisplatin (CisPt) (**a**) and the effects on MDA-MB-231 cells are reported to doxorubicin (DOX) (**b**).

A flow cytometry method was used to analyze the effect induced by the studied compounds on the cell cycle of the HT-29 tumor cells compared to the effect induced by CisPt, and on the cell cycle of the MDA-MB-231 tumor cells using DOX as a positive control (Figure 6).

The treatment of the HT-29 cells for 24 h with CisPt 10 μM induced a decrease in the G0/G1 phase from 54% to 21%, accompanied by an increase in the S phase of the cell cycle to 43.5% compared to the untreated cells (21.2%). The oxadiazoles **3a–d** did not significantly alter the proportion of G0/G1 phase cells compared to untreated cells, while increasing the number of S phase cells. The compound **3e** caused a different effect, augmenting the G0/G1 phase accompanied by a decrease in the S phase.

The flow cytometry analysis on the cell cycle of the MDA-MB-231 tumor cells showed a high percentage of G0/G1 phase (68.9%). The treatment with DOX 10 μM induced a synchronization of the S and G2+M phases, registering an increase in the S phase (42.4%) versus the untreated cells (5.1%), accompanied by an increase in the G2+M phases (43.9%) versus 26% in the untreated cells. The analyzed compounds determined the arrest of the cells in the G0/G1 phase, accompanied by a decrease in the S and G2+M phases. The compound **3e** had the greatest impact of the oxadiazoles series, increasing the number of cells in G0/G1 and significantly reducing those in the S phase.

### 2.3. Daphnia Magna Toxicity Assay

The *Daphnia magna* (*D. magna*) bioassay results are summarized in Table 2. After 24 h of exposure, the compounds **3b**–**e** induced at all the tested concentrations a lethality rate (L%) lower than 50%, whereas **3a** induced an L% of 55% at the highest concentration. Due to the obtained results, the median lethal concentration (LC50) was calculated only for the compounds **3a**, **2a** and **2b**. Although the LC50 for **2b** was slightly lower than **2a**, the 95%CI of both compounds suggest a similar biological response.

After 48 h of exposure, all the newly tested compounds exhibited a significantly higher toxicity on *D. magna*. The LC50 value was not calculated for **3a** and **3b** because they induced an L% between 55 and 85% at all the concentrations. In the case of the compounds **3c–e,** the calculated LC50 values are between 2.34 and 11.5 µM.

### 2.4. Prediction of the Molecular Mechanism of Action and Toxicity

#### 2.4.1. PASS Prediction

The prediction of an activity spectra for substances (PASS) is an algorithm that predicts a large panel of biological activities of a given molecule using its structure as input data, and yields a probability to be active (Pa) and a probability to be inactive (Pi) for each target [38]. Each compound’s target profile was manually analyzed, the relevant oncotargets were selected, and the corresponding Pa values are presented in Table 3.

The Pa values are an indication of the possibility that a compound interacts with a certain biological target, but not for the potency of the compound. The Pa values indicate the inhibition of STAT transcription factors, especially STAT3, as the most probable mechanism for the anti-proliferative effects of the compounds **3a–e**. The results for the compounds **3d** and **3a** indicate that the (phenylsulfonyl)methyl substitution reduces the probability to inhibit STAT3 compared to the phenylthiomethyl substitution.

#### 2.4.2. Structural Similarity Analysis

**Figure 7:**
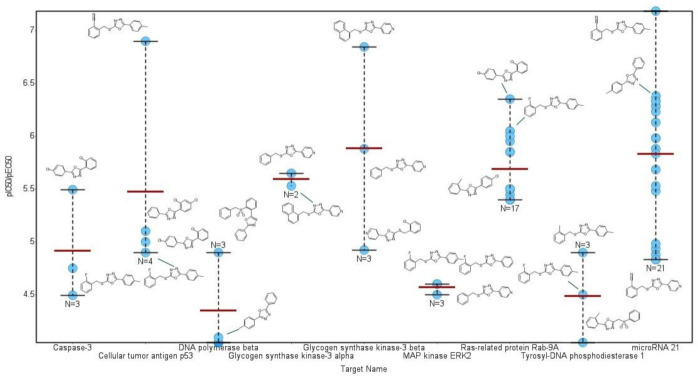
Structures and negative log of potency measurement for compounds structurally similar with the target structures **3a–e**. The compounds were extracted from the ChEMBL database and share a similarity over 50% with the new synthesized compounds. The red lines represent the average value for each target.

The similarity search on ChEMBL database returned 27 analog compounds, all sharing a 1,3,4-oxadiazole central scaffold. The highest degree of structural similarity (65.00%) was observed for the compound CHEMBL485773. The results highlight the originality of the new synthesized compounds. Depending on their structure, the ChEMBL compounds are registered to interact with various human targets. The most frequent targets are represented by microRNA 21 (77.78%), Ras-related protein Rab-9A (62.96%), Niemann–Pick C1 protein (59.26%), survival motor neuron protein (48.15%), and 15-hydroxyprostaglandin dehydrogenase (40.74%). In Figure 7 the corresponding pIC50 or pEC50 values are represented for the most relevant oncological target registered for the similar compounds.

The target profile of the chemically similar compounds indicates the interaction with microRNA 21 (miR-21), Ras-related protein Rab-9A, glycogen synthase kinase-3 beta, and cellular tumor antigen p53, as the most probable interaction targets for the new synthesized compounds **3a–e**.

#### 2.4.3. Predicted Acute Rat Toxicity

For all the compounds, the predicted results fall in the applicability domain of the application. The predicted median lethal dose (LD50) of the new compounds after oral and intravenous (IV) administration on rats are presented in Table 4, and they indicate a relatively low degree of toxicity. All the new oxadiazole derivatives are predicted to be less toxic than their corresponding hydrazides synthesis precursors.

## 3. Discussion

A series of new 2,5-diaryl/heteroaryl-1,3,4-oxadiazoles were synthesized and evaluated for their anticancer potential on two standardized cell lines, HT-29 and MDA-MB-231. At 10 μM, all the compounds reduced the cell viability after 24 h of exposure, inducing apoptosis and perturbation of the cell cycle. The cell line MDA-MB-231 proved to be more sensitive to the compounds’ action than HT-29.

The predictive studies using the PASS application indicated the inhibition of the STAT3 transcription factor as the most probable anticancer mechanism. Recent evidence shows that the 1,3,4-oxadiazole scaffold is frequently used in the structure of STAT3 inhibitors active against various cancer cells [39,40,41]. STAT3 is closely related to the occurrence of cancers and is an attractive therapeutic target for oncology and drug development. It acts in the regulation of many cellular events involving cell proliferation, differentiation, apoptosis and angiogenesis [42]. N-[2-(1,3,4-oxadiazolyl)]-4-quinolinecarboxamide, also known as STX-0119, is structurally similar to the **3a–e** compounds and functions as a STAT3 dimerization inhibitor [43]. HJC0123 was developed based on the structure of STX-0119, but it does not contain the oxadiazole scaffold. When administered in the MDA-MB-231 cells, the compound blocked the phosphorylation of STAT3, reduced the cells’ viability, promoted apoptosis, and increased the proportion of S-phase cells while reducing the number of cells in G0/G1 [44]. The effects of HJC0123 in the MDA-MB-231 cells are similar with those observed for compound **3e**. In contrast to MDA-MB-231 tumor cell lines, STAT3 plays no major role in the colon carcinoma cell line HT-29 [45] and it could explain the lower effects of the new compounds on this cell line.

The structural similarity analysis indicated miR-21 as a highly probable target for the new compounds. MiR-21 is considered an oncomir because it is one of the most frequently up-regulated miRNA in a wide type of cancers. MiR-21 is overexpressed in the MDA-MB-231 cell line close to four-fold compared with the non-tumorigenic MCF-10A cell line. The knockdown of miR-21 suppressed the cell growth and proliferation of the MDA-MB-231 cells [46]. The levels of miR-21 are also significantly higher in the HT-29 cells and promote cell proliferation and migration [47].

The results of the predictive studies using the PASS application and the structural similarity analysis indicated STAT3 and miR-21 as the most probable pharmacological targets for the new compounds **3a–e**, but also that these compounds may have multitarget activities. This is suggested also by the significant toxic effects registered in the *D. magna* 48 h assay.

The chemical diversity of the **3a–e** structures, and the limited number of tested compounds, limits the development of structure activity relationships. The compound **3e** presented the best anticancer profile of the series, probably due to the presence of the pyridine ring next to the oxadiazole structure. The promising effect of the compound **3e**, especially on the MDA-MB-231 cell line, a triple-negative breast cancer line, motivates future studies to improve the anticancer profile and to reduce the toxicological risks. It is worth noting that **3e** produced a low toxic effect in the *D. magna* 24 h assay and the predictive studies on rat acute toxicity suggest a low degree of toxic risks.

## 4. Materials and Methods

### 4.1. Analytical Procedures

The melting points (m.p.) were measured in open capillary tubes on an Electrothermal 9100 apparatus and are uncorrected. The ^1^H-NMR and ^13^C-NMR spectra were recorded on a Gemini 300 BB instrument (Varian, Palo Alto, CA, USA) at room temperature, operating at 300 MHz for ^1^H and 75.075 MHz for ^13^C. The chemical shifts were recorded as δ values in ppm units downfield to tetrametylsilane (TMS) used as internal standard, and CDCl_3_ and DMSO-d6 as solvents. The coupling constants values (*J*) are reported in hertz (Hz) and the splitting patterns are abbreviated as follows: s, singlet; d, doublet; t, triplet; q, quartet; and b, broad. The carbons not attached to any protons are presented as Cq, while those attached to a hydrogen atom are designated as CH.

The IR spectra were recorded on a FT/IR-4200 spectrometer (JASCO, Tokyo, Japan) with an ATR PRO450-S accessory at a resolution of 4 cm^−1^. The elemental analyses were performed on a Perkin–Elmer 2400 Series II CHNS/O Elemental Analyzer (Shelton, CT, USA).

### 4.2. Synthesis Procedures

All the chemicals and reagents were purchased from commercial suppliers and used without purification, unless otherwise noted.

#### 4.2.1. Synthesis of 2-[(benzenesulfonyl)methyl]benzoic acid (**1d**)

To a solution of 2-(phenylthiomethyl)benzoic acid (**1a**) (0.02 mol) in glacial acetic acid (100 mL), 20 mL 30% aqueous hydrogen peroxide was added dropwise. The mixture was heated for 2 h and then left overnight at room temperature. The reaction mixture was diluted with water and extracted with chloroform. The separated organic phase was dried over sodium sulfate and then concentrated under reduced pressure. The crude product was recrystallized from ethanol.

White solid. Yield 93%, m.p. 154–155 °C. IR (cm^−1^). ^1^H-NMR (300 MHz, CDCl_3_, δ ppm, *J* Hz): 8.86 (bs, 1H); 8.05 (dd, 1H, 1.4, 7.5); 7.64 (dd, 2H, 1.4, 7.5); 7.59 (tt, 1H, 1.4, 7.2); 7.55 (td, 1H, 1.4, 7.5); 7.49 (m, 1H); 7.46 (m, 2H); 7.35 (dd, 1H, 1.4, 7.5); 5.10 (s, 2H). ^13^C-NMR (75.075 MHz, CDCl_3_, δ ppm): 171.88 (C-7); 137.90 (C-9); 129.97 (C-2); 129.45 (C-1); 129.04 (C-3); 128.98 (C-11, C-13); 128.62(C-10, C-14); 133.88(C-4 or C-5); 133.74 (C-12); 133.02 (C-5 or C-4); 131.86 (C-6); 59.46 (C-8). Elemental analysis calculated for C_14_H_12_O_4_S (276.31 g/mol): C 60.86%, H 4.38%, S 11.60% and found: C 60.94%, H 4.28%, S 11.69%.

#### 4.2.2. General Procedure for the Synthesis of the 1,3,4-oxadiazoles Derivatives (**3a-e**)

An equimolar mixture of benzoyl hydrazine (**2a**, 0.01 mol) or isonicotinic hydrazide (**2b**, 0.01 mol) and the appropriate aromatic acid (**1a–d**) (0.01 mol) in phosphorus oxychloride (45 mL) was refluxed for 9 h. The reaction mixture was slowly poured onto crushed ice and kept overnight. The solid thus separated out was filtered and washed with water, dried under vacuum and recrystallized from an appropriate solvent.

#### 4.2.3. 2-Phenyl-5-[2-(phenylsulfanylmethyl)phenyl]-1,3,4-oxadiazole (**3a**)

White solid. Yield 69%, m.p. 111–112 °C. ^1^H-NMR (300 MHz, CDCl_3_, δ ppm, *J* Hz): 8.03 (dd, 7.7, 1.6, 2H, H-20, H-24); 7.95 (ddd, 1H, H-7); 7.42–7.49 m (3H, H-21, H-22, H-23); 7.27–7.35 m (3H; H-8, H-9, H-10); 7.22 td (2H, H-14, H-18); 7.07–7.15 m (3H, H-15, H-16, H-17): 4.64 (s, 2H, H-12). ^13^C-NMR (75.075 MHz, CDCl_3_, δ ppm): 164.39 (C-2 or C-5); 164.26 (C-2 or C-5); 138.07 (Cq); 135.60 (Cq); 131.90 (CH); 131.61 (2CH); 131.44 (CH); 131.39 (CH); 129.51 (CH); 129.23 (2CH); 128.90 (2CH); 127.75 (CH); 127.13 (2CH); 127.02 (CH); 123.97 (CH); 122.84 (CH); 38.30 (C-12). Elemental analysis calculated for C_21_H_16_N_2_OS (344.44 g/mol): C 73.23%, H 4.68%, N 8.13%, S 9.31% and found: C 73.31%, H 4.60%, N 8.20%, S 9.24%.

#### 4.2.4. 2-Phenyl-5-[2-(p-tolylsulfanylmethyl)phenyl]-1,3,4-oxadiazole (**3b**)

White solid. Yield 68%, m.p. 116–117 °C. ^1^H-NMR (300 MHz, CDCl_3_, δ ppm, *J* Hz): 8.11 (dd, 7.4, 1.5, 2H, H-20, H-24); 8.01 (dd, 7.0, 2.2, 1H, H-7); 7.48–7.58 (m, 3H, H-8, H-9, H-10); 7.40 (t, 6.5, 2H, H-21, H-23); 7.31 (t, 6.5, 1H, H-22); 7.17 (d, 8.1, 2H, H-15, H-17); 6.98 (d, 8.1, 2H, H-14, H-18); 4.66 (s, 2H, H-12); 2.22 (s, 3H, CH_3_). ^13^C-NMR (75.075 MHz, CDCl_3_, δ ppm): 164.21 (C-2 or C-5); 164.16 (C-2 or C-5); 138.32 (Cq); 137.20 (Cq); 137.19 (Cq); 132.38 (CH); 132.28 (CH); 131.73 (CH); 131.34 (CH); 131.21 (CH); 129.51 (2CH); 129.32 (CH); 129.07 (2CH); 127.49 (CH); 126.96 (2CH); 123.83 (Cq); 122.64 (Cq); 38.70 (C-12); 21.01 (CH_3_). Elemental analysis calculated for C_22_H_18_N_2_OS (358.47 g/mol): C 73.72%, H 5.06%, N 7.81%, S 8.94% and found: C 73.81%, H 4.98%, N 7.72%, S 9.01%.

#### 4.2.5. 2-[2-[(4-Chlorophenyl)sulfanylmethyl]phenyl]-5-phenyl-1,3,4-oxadiazole (**3c**)

White solid. Yield 60%, m.p. 126–128 °C. ^1^H-NMR (CDCl_3_, δ ppm *J* Hz): 8.06 (dd, 1.6, 7.5, 2H, H-20, H-24); 7.97 ddd (1H, H-7); 7.47–7.50 m (3H, H-21, H-22, H-23); 7.38–7.35 m (2H; H-9, H-10); 7.26 (dd, 1H, H-8); 7.16 (d, 8.8, 2H, H-14, H-18); 7.10 (d, 8.8, 2H, H-15, H-17); 4.65 (s, 2H, H-12); ^13^C-NMR (CDCl_3_, δ ppm): 164.42 (C-2(5)); 164.16 (C-5(2)); 137.85 (C-13); 134.03 (Cq); 133.73 (Cq); 133.21 (2CH); 131.98 (CH); 131.43 (2CH); 129.53 (CH); 129.26 (2CH); 129.04 (2CH); 127.91 (CH); 127.16 (2CH); 123.92 (Cq); 122.87 (Cq); 38.56 (C-12). Elemental analysis calculated for C_21_H_15_ClN_2_OS (378.88 g/mol): C 66.57%, H 3.99%, N 7.39%, S 8.46% and found: C 66.68%, H 3.82%, N 7.48%, S 8.32%.

#### 4.2.6. 2-[2-(Benzenesulfonylmethyl)phenyl]-5-phenyl-1,3,4-oxadiazole (**3d**)

White solid. Yield 59%, m.p. 195–196 °C. ^1^H-NMR (300 MHz, CDCl_3_, δ ppm, *J* Hz): 7.97 (dd, 8.0, 2.4, 2H, H-14, H-18); 7.77 (dd, 7.1, 1.8, 1H, H-7); 7.00–7.60 (m, 11H, H-arom); 5.26 (s, 2H, H-12). ^13^C-NMR (75.075 MHz, CDCl_3_, δ ppm): 164.05 (C-2 or C-5); 163.46 (C-2 or C-5); 138.07 (Cq); 134.03 (CH); 133.48 (CH); 132.09 (CH); 131.60 (CH); 129.46 (CH); 129.27 (2CH); 129.09 (CH); 128.87 (CH); 128.72 (2CH); 128.69 (CH); 127.99 (Cq); 127.02 (2CH); 124.37 (Cq); 123.53 (Cq); 59.38 (C-12). Elemental analysis calculated for C_21_H_16_N_2_O_3_S (376.44 g/mol): C 67.01%, H 4.28%, N 7.44%, S 8.52% and found: C 67.09%, H 4.19%, N 7.38%, S 8.60%.

#### 4.2.7. 2-[2-(Phenylsulfanylmethyl)phenyl]-5-(4-pyridyl)-1,3,4-oxadiazole (**3e**)

White solid. Yield 70%, m.p. 130–132 °C. ^1^H-NMR (300 MHz, DMSO-d6 + CDCl_3_ 3:1, δ ppm, *J* Hz): 8.84 (d, 5.8, 2H, H-21, H-23); 8.07 (td, 4.0, 2.0, 1H, H-7); 8.03 (d, 5.8, 2H, H-20, H-24); 7.39–7.49 (m, 3H, H-8, H-9, H-10); 7.13–7.26 (m, 5H, H-14, H-15, H-16, H-17, H-18); 4.71 (s, 2H, H-12). ^13^C-NMR (75.075 MHz, DMSO-d6 + CDCl_3_ 3:1, δ ppm): 164.50 (C-2 or C-5); 161.87 (C-2 or C-5); 149.90 (C-21, C-23); 137.56 (Cq); 135.07 (Cq); 131.63 (CH); 131.14 (CH); 131.03 (CH); 129.97 (2CH); 129.50 (CH); 128.64 (2CH); 127.73 (CH); 126.37 (CH); 121.70 (CH); 120.41 (2CH); 36.54 (C-12). Elemental analysis calculated for C_20_H_15_N_3_OS (345.43 g/mol): C 69.54%, H 4.38%, N 12.16%, S 9.28% and found: C 69.48%, H 4.46%, N 12.26%, S 9.19%.

### 4.3. Anticancer Evaluation

#### 4.3.1. Reagents

Cisplatin (CisPt), doxorubicin (DOX), and dimethyl sulfoxide (DMSO) were purchased from Sigma-Aldrich (St. Louis, MO, USA). The stock solutions were prepared by dissolving the compounds in a minimum amount of DMSO and kept at −20 °C. The working solutions were prepared before each experiment from the stocks and the culture medium. Annexin V-FITC/PI Apoptosis Detection Kit for flow cytometry was purchased from BioVision Inc., Milpitas, CA, USA. Cycletest Plus DNA Reagent Kit was provided by BD Biosciences (Becton Dickinson, USA).

#### 4.3.2. Cell Culture and Treatments

Human cancer cell lines HT-29 (colon adenocarcinoma) and MDA-MB-231 (breast adenocarcinoma) were purchased from American Type Culture Collection (ATCC). Adherent cells were routinely maintained in culture in Dulbecco’s modified Eagle medium/nutrient mixture F-12 (DMEM:F12) medium added by 2 mM of L-glutamine, 10% fetal bovine serum, 100 units/mL penicillin, 100 μg/mL streptomycin (Sigma-Aldrich, St. Louis, Mo, USA) and incubated at 37 °C in 5% CO_2_ humidified atmosphere. After 24 h, adherent cells were treated with different concentrations of the compounds for different periods of time. Cell treatments of compounds, CisPt and DOX were carried out using concentrations of 200, 100, 50, 25, 12.5 and 6.25 μM of the drug. Then cells were detached with a nonenzymatic solution of phosphate-buffered saline (PBS)/1 mM EDTA, washed twice in PBS.

#### 4.3.3. Cytotoxicity Assay

All assays were performed in triplicate in 96-well microtiter plates with flat bottom (Falcon), using CellTiter 96 Aqueous One Solution Cell Proliferation Assay (Promega), an MTS (3-(4,5-dimethylthiazol-2-yl)-5-(3-carboxymethoxyphenyl)-2-(4-sulfophenyl)-2*H*-tetrazolium)-based colorimetric assay. Briefly, 1 × 10^4^ cells/wells were cultured in 100 μL for 24 h, culture supernatants were discarded, and then cells were treated for 24 h and 48 h with increasing concentrations of drugs. After the end of the incubation time, 20 μL reagent containing a) MTS, and b) phenazine ethosulfate (PES) were added in each well. PES has a high chemical stability that allows it to bind to MTS and form a stable solution. After adding the coloring solution, plates were incubated for 4 h at 37 °C, with mild agitation every 15 min. The method relies on the ability of metabolically active cells to reduce MTS, a yellow tetrazolium salt to the colored formazan that is soluble in the culture medium. The reduction in the tetrazolium compound to formazan was spectrophotometrically measured at λ = 492 nm, using a Dynex plate reader (DYNEX Technologies-MRS). The percentage of viability compared to untreated cells (considered 100% viable) was calculated based on the absorbance (Abs) values as follows:

Cell viability (%) = (Abs treated cells − Abs culture medium)/(Abs untreated cells − Abs culture medium) × 100,(1)

Cell viability data were expressed as the mean values ± standard deviations (SD) of the experiments. Data were obtained in triplicates (*n* = 3), averaged and expressed as mean ± SD.

#### 4.3.4. Apoptosis Analysis

The apoptosis assay was carried out using the Annexin V-FITC Kit and the manufacturer’s protocol from BD Biosciences. The 5 × 10^5^ cells/mL treated and untreated were suspended in cold binding buffer and stained simultaneously with 5 μL FITC-Annexin V (green fluorescence) and 5 μL PI in a dark at room temperature for 15 min. The percentages of apoptotic cells were determined by double staining with Annexin V-FITC/ PI. In each tube was added 400 μL of Annexin V binding buffer and the 5000 cells/sample were collected using FACSCantoII flow cytometer (Becton Dickinson—BD) and the analysis was performed using DIVA 6.2 software in order to identify early apoptosis (Annexin+/PI−), late apoptosis (Annexin+/PI+) and necrosis (Annexin−/PI+) [48].

#### 4.3.5. Cell Cycle Analysis

The assay was carried out using Cycletest Plus DNA Reagent Kit and the manufacturer’s protocol from BD Biosciences. Previously fixed cells (5 × 10^5^) were washed twice in PBS and cell pellets were resuspended in PBS. The probes were kept in the dark and at 4 °C until data acquisition by flow cytometry using a FACSCantoII flow cytometer (Becton Dickinson—BD). The analysis was performed using ModFIT software in order to estimate the DNA index (DI) and progression through cell cycle phases [49].

### 4.4. Daphnia Magna Toxicity Assay

*D. magna* Straus was maintained parthenogenetically at ‘Carol Davila’ University (Department of Pharmaceutical Botany and Cell Biology). The culture was maintained at 25 °C, a photoperiod of 16 h/8 h light/dark cycle. Prior to the determination, young daphnids were selected according to their size and maintained for 24 h in artificial medium. The bioassay was performed on 10 daphnids/replicates in tissue culture plates with 12 wells (Greiner Bio-One) according to the protocol described in our previous studies [50,51]. For each compound, six concentrations were tested, ranging from 5 to 128 µM. The hydrazides **2a** (20–411 µM) and **2b** (20–394 µM) were used as positive controls, and a 1% DMSO solution as a negative control. The concentrations were selected based on the solubility and a pre-screening assay. The final volume/well was 4 mL, and the lethality was recorded at 24 and 48 h of exposure. All determinations were performed in duplicate. The 95% confidence intervals (95%CI) for LC50 values were also calculated using the least square fit method. All calculations were performed using GraphPad Prism v 5.1 software.

### 4.5. Prediction of the Molecular Mechanism of Action and Toxicity

#### 4.5.1. PASS Prediction

A virtual screening was performed using the computer program PASS (prediction of activity spectra for substances), a software product designed to evaluate the general biological potential drug-like molecules. The compounds were inputted in PASS as mol files and the results were analyzed if the Pa values were above the corresponding Pi values. The resulted biological targets were manually selected based on their anticancer treatment potential.

#### 4.5.2. Structural Similarity Analysis

A similarity search was performed on the ChEMBL database for each compound **3a–e** using a 50% threshold. The resulting structures were extracted together with their assayed activities on human targets [52]. The entries were filtered using DataWarrior v5.2.1 software [53] to remove compounds with inexact potency values and to merge duplicate structures into single entries with calculated average pIC50 or pEC50 values expressed as mol/L (M).

#### 4.5.3. Prediction of the Compounds’ Toxicity

The freely available program GUSAR was used to predict the LD50 values of the new compounds after oral and intravenous administration on rats [54].

## 5. Patents

Patent application a202000446: Camelia Elena Stecoza, George Mihai Nitulescu, Mirela Antonela Mihaila, Marinela Bostan, Constantin Draghici, Miron Teodor Caproiu, 2-Aryl(heteroaryl)-5-[2-(phenylthiomethyl)phenyl]-1,3,4-oxadiazole derivatives, a pharmaceutical composition containing them and their use as antitumor agent, published in RO-BOPI, 11/2020 from 27 November 2020.

## Figures and Tables

**Table 1:** Apoptosis of HT-29 cells and MDA-MB-231 cells induced by 24 h treatment with the tested compounds **3a–e** and **2a–b**.

	HT-29 Cells			MDA-MB-231 Cells		
	Early Apoptosis (%)	Late Apoptosis (%)	Total Apoptosis (%)	Early Apoptosis (%)	Late Apoptosis (%)	Total Apoptosis (%)
Control	4.8	1.1	5.9	4.2	0.5	4.7
CisPt ^1^	16.5	4.2	20.7	-	-	-
DOX ^1^	-	-	-	11.4	2.3	13.7
**3a**	23.5	2.2	25.7	41.7	8.0	49.7
**3b**	21.7	4.8	26.5	40.5	4.7	45.2
**3c**	6.4	3	9.4	40.8	5.0	45.8
**3d**	16.5	2.7	19.2	44.3	6.6	50.9
**3e**	43.9	7.3	51.2	51.8	10.9	62.7
**2a**	53.0	12.7	65.7	45.9	12.9	58.8
**2b**	44.8	6.8	51.6	55.5	17.3	72.8

^1^ positive controls cisplatin (CisPt) for HT-29 cells and doxorubicin (DOX) for MDA-MB-231 cells.

**Table 2:** Daphnia magna bioassay: 24 h and 48 h values, 95% confidence intervals for newly synthetized compounds and their starting materials.

Compound	24 h		48 h	
	LC50 (µM)	95%CI of LC50 (µM)	LC50 (µM)	95%CI of LC50 (µM)
**3a**	115.8	43.6 to 307.4	ND*	ND*
**3b**	ND*	ND*	ND*	ND*
**3c**	ND*	ND*	11.5	ND*
**3d**	ND*	ND*	2.34	ND*
**3e**	ND*	ND*	3.5	2.0–7.3
**2a**	332.5	201.1–549.8	35.9	23.0–56.1
**2b**	296.0	205.3–426.7	21.8	11.5–41.1

ND*—not determined due the results obtained.

**Table 3:** The probability of compounds **3a–e** to be active (Pa) as predicted by PASS analysis.

Target	3a	3b	3c	3d	3e
Transcription factor inhibitor	0.58	0.62	0.55	0.32	0.58
Transcription factor STAT inhibitor	0.61	0.64	0.61	0.42	0.65
Transcription factor STAT3 inhibitor	0.55	0.58	0.56	0.26	0.54
JAK2 expression inhibitor	0.40	0.33	0.40	0.33	0.22
Focal adhesion kinase inhibitor	0.23	0.22	0.22	0.24	0.27
Focal adhesion kinase 2 inhibitor	0.35	0.34	0.34	0.36	0.38
MAP3K5 inhibitor	0.28	0.26	-	-	0.29
Vascular endothelial growth factor 1 antagonist	0.27	0.22	0.22	0.30	0.30

**Table 4:** The predicted toxicity of the compounds **3a–e** and **2a–b** expressed as median lethal doses (LD50) on rats using the GUSAR application.

Rat Acute Toxicity	3a	3b	3c	3d	3e	2a	2b
Oral LD50 (mg/kg)	1970	1977	1317	1417	1924	967	900
Oral LD50 Class	4	4	4	4	4	4	4
IV LD50 (mg/kg)	321.1	289.6	253.3	383.0	298.8	135.7	256.1
IV LD50 Class	5	4	4	5	4	4	4

## Data Availability

The datasets used and/or analysed during the current study are available from the corresponding author on reasonable request.
